# Thank You to Our 2025 Peer Reviewers

**DOI:** 10.1029/2026GH001928

**Published:** 2026-04-08

**Authors:** Thanh H. Nguyen, Assaf Anyamba, John Balbus, Kai Chen, Sagnik Dey, Meredith Franklin, Tzung‐May Fu, Meng Gao, Sunny Jiang, Yang Liu, Adina Paytan, Moiz Usmani, Steve Yim

**Affiliations:** ^1^ University of Illinois Urbana‐Champaign Urbana IL USA; ^2^ Goddard Earth Sciences Technology Center University of Maryland Baltimore County Baltimore MD USA; ^3^ Milken Institute School of Public Health The George Washington University Washington DC USA; ^4^ Department of Environmental Health Sciences Yale School of Public Health New Haven CT USA; ^5^ Indian Institute of Technology Delhi Centre for Atmospheric Sciences New Delhi Delhi India; ^6^ Department of Statistical Sciences University of Toronto Toronto ON Canada; ^7^ School of Environmental Science and Engineering Southern University of Science and Technology Shenzhen China; ^8^ Department of Geography Hong Kong Baptist University Hong Kong China; ^9^ University of California, Irvine Irvine CA USA; ^10^ Emory University Rollins School of Public Health Atlanta GA USA; ^11^ University of California, Santa Cruz Santa Cruz CA USA; ^12^ The University of Alabama at Birmingham Birmingham AL USA; ^13^ Asian School of the Environment Nanyang Technological University Singapore Singapore

**Keywords:** editorial, peer review

## Abstract

- The editors thank the 2025 peer reviewers

Peer‐review is the foundation and the safeguard of scientific research. Without the dedication of our reviewers, the journal would not have been successful. In 2025, 334 reviewers completed the review for manuscripts submitted to GeoHealth. Our reviewers are from all continents except Antarctica. Besides reviewers from North America, China, Europe, and China, we started to have reviewers from India, Latin America, and Africa. GeoHealth editorial board is committed to expanding the readership, authorship, and reviewership to other countries. If you have already reviewed for us, no matter where or who you are, we hope you and your colleagues will consider GeoHealth a home for your work.

Individuals in *italics* provided three or more reviews for GeoHealth during the year.

Abhishek Anand

Abinaya Sekar

Abu Mohammed Naser Titu

Abu Ubayeda Ibnal Zarrah

Aditya Vyas

Aidan Ocampo

Aika Davis

Ai‐Ling Jiang

Ainslee Wong

Alberto Bellin

Alex Perkins

Alexandros Angelakis

Ali Berkay Avci

Ali Sattari

*Ali Shafqat Akanda*

*Amanda Giang*

*Amanda Hoffman‐Hall*

Amit Raysoni

*Amy Fitch*

Anais Teyton

*Andrea Buchwald*

Andrea Pozzer

Ann Ojeda

Ann Wylie

Anquan Xia

Arman Oliazadeh

*Arnab Dey*

Arthit Phosri

Arthur Stem

Arundhati Bakshi

Ashraf Dewan

Assaf Anyamba

August Harrell

*Bailey Magers*

Balaji Ramesh

Ben Armstrong

Benjamin Bostick

Bin Han

Bryttani Wooten

Carl Malings

Carolina Vieira

Cassandra O'Lenick

Changwoo Han

Chanud Yasanayake

Charlie Chesney

Charlie Zhang

Chiara Arrighi

Chloe Brimicombe

Chris Fook Sheng Ng

Christina Kellogg

Christopher Emrich

Christopher Tessum

Chunyu Guo

C. J. Gabbe

Cong Liu

Craig Baker‐Austin

Daniel Goldberg

Daniel Horton

Daniel Kilpatrick

Daniel Kollath

*Danlu Zhang*

Di Wu

*Donald Smith*

Dorothy Lsoto

Ebba Malmqvist

Edmund Yamba

Eleanor Rogan

Elizabeth Berg

Emily Goddard

Eric Bump

Eric Coker

Ezequiel Zamora

Fahmida Parvin

Fangxia Shen

Fengchao Liang

Francis Oloo

*Gabriel Filippelli*

Gabriel Lade

Gajapriya Gajapathi

Gayle Tyree

Gerard FitzGerald

Guoxing Li

Haitao Wei

Hans Brenna Schjønberg

Hansong Zhu

Haoran Yu

Haosu Tang

Harshit Gujral

Hayon Choi

Heather Holmes

Hedayatullah Ehsan

Helene Ueno

*Heming Bai*

Hien Ngo

Hoda Hallaji

Holly Nowell

Holly Puglis

Howard Chang

Hua Hao

Huizhong Shen

Hunter Quon

Iain Lake

Isaac Addo

Isabel Byrne

Isabelle Bunge

Jabeen Taiba

Jacqueline Barkoski

Jahred Liddie

James King

Javad Shafiei Shiva

Jean‐Marie Robine

*Jennifer Head*

Jennifer Horney

Jennifer K. Vanos

*Jennifer Richmond‐Bryant*

Jennifer Stowell

Jiachen Zhang

Jiangjiang Zhang

Jianjun Xiang

Jianmin Ma

Jiayu Zhang

Jie Zhang

JinFeng Wang

Jinyan Wang

John S. Ji

Jonas Schmidt‐Chanasit

Jonathan Buonocore

Jonathan Levy

Jonathan Patz

Jonathan W. Chipman

Joseph Puthussery

Josh Colston

Josiah Kephart

Judith O'Neil

Jun Meng

Jun Yang

Juwel Rana

*Karen Holcomb*

Karn Vohra

Kate Beach

Keita Wagatsuma

Keith Bein

Keith Loftin

Keith Spangler

Keyong Huang

Kien Dang

Kirsten Wiens

Kone Zeinab

Koushan Mohammadi

Kristen Aiemjoy

Kristen Cowan

Kun Liu

Kwun Yip Fung

Lara Schwarz

Laura Andrea Rodriguez‐Villamiza

Lauren Broyles

Lei Huang

Li Li

*Lingzhi Chu*

Loki Snyman

Louie Kroll

Lourdes Vera

Luis Chaves

Makoto Kelp

Marcos Quijal‐Zamorano

Mark Laidlaw

Mark Paton

Martha Dellar

Martijn Schaap

Mary Finley‐Brook

Marya Lieberman

Matthew Gonnerman

Matthew Polizzotto

Matthew Tully

Mattia Santoro

*Mayank Gangwar*

Megan Coffer

Megan E. Meuti

Melissa Fiffer

Melissa Furlong

Meng Gao

Meng Wang

Mercedes Pascual

Michael Ashley Stein

Michael Cheeseman

Michael McGlade

Michelle Kondo

Miklas Scholz

Ming Ye

*Minghao Qiu*

Mitchell Manware

Mohammad Sofi Ullah

Mohammad Washeem

*Moiz Usmani*

Mostafijur Rahman

Muhammad Fawad

*Muhammad Nawaz*

Munazza Fatima

Muye Ru

Nadav Peleg

*Nancy Johnston*

Nathan Pavlovic

Nathan Phillips

Naveen Joseph

Nicolas Zanetta Colombo

Ningrui Liu

P. Gopinathan

Pallavi Pant

Patricia Landaverde

Paula Andrea Fonseca

Phong Le

*Pin Wang*

Prince Eliot Sounga Bandzouzi

Qi Meng

Qi Zhao

Qiang Guo

Qiao Zhu

Qingyang Zhu

Qinqin Kong

Qirui Zhong

*Qurat Ul An Sabir*

Rachel Connolly

Rachel Edie

Rachel Yuen Sum Tam

Rafael Lopes

Ramesh Dhiman

Ravinder Arigela

Rawil Fakhrullin

Ricardo Guimarães

Richard Damoah

Riyang Liu

Robbie Parks

Ruijie Zeng

Ruud Janssen

Ryan Harp

Sadie J. Ryan

Sahar Zinatloo‐Ajabshir

Sally Jahn

Sam Heft‐Neal

Sana Arshad

Saowanee Norkaew

Sara Maria Barbani

Sara McLafferty

Sarah Rothenberg

Saravanan Arunachalam

Saroj Mishra

Saumya Singh

Sean Young

*Serap Arsal Yıldırım*

Sewwandi Bandara

Shaul Schreiber

Sheryl Magzamen

Shin‐Hong Shiao

Shuaiyin Chen

Sidharth Sivaraj

Sishir Dahal

Sneha Gautam

*Song Liu*

Sonia Kreidenweis

Soo‐Yeon Kim

Sophia Ryan

Sotiria Koloutsou‐Vakakis

Srinivasa Rao Mutheneni

Steve Yim

Sujung Lee

Sumaira Zafar

Sumil Thakrar

Sung‐mok Jung

*Sunil Kumar*

Susan Rumisha

Sylvia Dee

*Szu Yu Lin*

Tabish Ansari

Taimoor Shah Durrani

Tao Hu

*Tao Huang*

Tao Xue

Tekleab Gala

Thanh Nhan Duc Tran

Tian Ma

Tiangang Yuan

Tingting Fang

Tingting Hu

Tingting Ye

Tobias Vogt

Trent Biggs

Tsz Shan Ma

Tuyet‐Mai Hoang

Tzung‐May Fu

Ufuoma Ovienmhada

Utsab Ghosal

Vaidyanathan Ambarish

Wanghuan Hao

Wangjian Zhang

Wataru Morioka

Wei Hu

Weiwei Chen

Wensu Zhou

*Xia Meng*

Xiao He

Xiao Lin

Xiaoxu Wu

*Xin Meng*

*Xueli Yang*

Xueying Zhang

Yaling Zeng

Yasar Burak Oztaner

*Yefu Gu*

Yehua Dennis Wei

Yeong Min Kim

Yi Guo

Yichen Wang

Ying Hu

*Yiqun Ma*

*Yog Aryal*

Youn Soo Jung

Youn‐Suk Son

Yu Li

*Yue Li*

Yue Lin

Yuhang Pan

Yun Hang

*Yusuf Jamal*

Yutong Zhong

Yuxuan Zhou

Yuzhou Wang

Zachary Calhoun

Zhanxiang Wang

*Zhaobin Sun*

Zhaoyuan Li

Zheng Xian

Zhengyi Cui

Zhenjie Zhao

Zhuohui Zhao

